# Sympathetic Blocks Provided Sustained Pain Relief in a Patient with Refractory Painful Diabetic Neuropathy

**DOI:** 10.1155/2012/285328

**Published:** 2012-02-06

**Authors:** Jianguo Cheng, Anuj Daftari, Lan Zhou

**Affiliations:** ^1^Department of Pain Management, Cleveland Clinic, 9500 Euclid Avenue, Cleveland, OH 44195, USA; ^2^Department of Physical Medicine and Rehabilitation, Metrohealth Medical Center, 2500 Metrohealth Drive, Cleveland, OH 44109, USA; ^3^Department of Neurology, Cleveland Clinic, 9500 Euclid Avenue, Cleveland, OH 44195, USA

## Abstract

The sympathetic nervous system has been implicated in pain associated with painful diabetic neuropathy. However, therapeutic intervention targeted at the sympathetic nervous system has not been established. We thus tested the hypothesis that sympathetic nerve blocks significantly reduce pain in a patient with painful diabetic neuropathy who has failed multiple pharmacological treatments. The diagnosis of small fiber sensory neuropathy was based on clinical presentations and confirmed by skin biopsies. A series of 9 lumbar sympathetic blocks over a 26-month period provided sustained pain relief in his legs. Additional thoracic paravertebral blocks further provided control of the pain in the trunk which can occasionally be seen in severe diabetic neuropathy cases, consequent to extensive involvement of the intercostal nerves. These blocks provided sustained and significant pain relief and improvement of quality of life over a period of more than two years. We thus provided the first clinical evidence supporting the notion that sympathetic nervous system plays a critical role in painful diabetic neuropathy and sympathetic blocks can be an effective management modality of painful diabetic neuropathy. We concluded that the sympathetic nervous system is a valuable therapeutic target of pharmacological and interventional modalities of treatments in painful diabetic neuropathy patients.

## 1. Introduction

Diabetic polyneuropathy is one of the most common forms of peripheral neuropathy. It afflicts patients of both type 1 and type 2 diabetes with an increased prevalence as the disease progresses [1–3]. Up to 50% of all diabetics with long-duration diabetes have polyneuropathy which is a major cause of morbidity and is associated with increased mortality. Up to 26% of diabetics develop painful diabetic neuropathy (PDN) with debilitating effects on quality of life [4–6]. Management of PDN remains an enormous challenge to both the patients and the clinicians as we have recently reviewed [7]. The current strategy includes mandatory glycemic control and pain control by pharmacological treatment with local anesthetic patches, anticonvulsants, tricyclic antidepressants, selective serotonin and noradrenalin reuptake inhibitors, and/or opioids. Spinal cord stimulation has been tested in a few studies involving a small number of highly selected patients who failed to respond to conservative treatments, with some degree of positive effects [8, 9]. However, the pain control of diabetic neuropathy remains a daunting challenge and the overall outcomes of the current management of diabetic neuropathy are not satisfactory.

Although diabetic polyneuropathy is clinically known for over a century, the pathophysiological mechanisms were only recently better understood. It is recognized that the microvascular dysfunction, secondary to chronic hyperglycemia and dyslipidemia, is a common pathophysiological basis of polyneuropathy and other microvascular complications with diabetes. There is also evidence that the sympathetic nervous system may play an important role in painful diabetic neuropathy. Circulating norepinephrine is higher in painful than painless diabetic neuropathy, and its concentration is correlated with the severity of neuropathic pain [10]. Thus, painful diabetic neuropathy is suggested to be associated with a relatively higher number of functioning sympathetic fibers that may contribute to pain. Damaged peripheral nerves became hyperexcitable through abnormal electrical connections that may have resulted in ephaptic transmission or “crosstalking” between sensory and sympathetic nerve fibers [11, 12]. Indeed, norepinephrine excited the ongoing ephaptic activity in damaged peripheral nerves through activation of alpha receptors [11]. Furthermore, patients with PDN had impaired sympathetically mediated vasoconstriction, contributing to inappropriate local blood flow regulation in these patients [13].

Based on these observations, we hypothesized that sympathetic nerve blocks may reduce pain associated with diabetic neuropathy by reducing sympathetic outflow and improving circulation. We tested this hypothesis in a patient with severe PDN refractory to multiple pain medications by treating him with lumbar and thoracic sympathetic blocks. The diagnosis of small fiber sensory neuropathy was based on clinical presentations and confirmed by skin biopsies. A series of 9 lumbar sympathetic blocks over a 26-month period provided sustained pain relief in his legs. Additional thoracic paravertebral blocks further provided control of his pain in the trunk from dermatomes T6 to L1, consequent to extensive involvement of PDN. These blocks significantly improved his quality of life over a period of more than two years.

## 2. Case Report

The patient is a 37-year-old right-handed Caucasian man who was in his usual state of health until December 2006 when he started to notice that his feet were cold, numb, and had a tingling sensation (described as pins and needles) from the ankles down. In a few weeks, the tingling sensation progressed up to the knees which remained stable for the next three months. In April 2007, he also noted the tingling sensation in the arms.

At time of his presentation to our pain clinic and neurology clinic in October 2007, he reported diffuse constant tingling sensation, mostly involving his arms, legs, and face, which was accompanied by sharp pains, mostly in the feet and distal legs. The pain was rated on average as an 8 on a numerical rating scale (NRS) (0 is no pain and 10 is most severe pain imaginable). He also reported episodic lower extremity allodynia to light touch and burning dysesthesia in his trunk and hands. These symptoms kept him up at night and disturbed his sleep. He began to experience symptoms of depression secondary to the relentless pain condition. He denies weakness. Physical examination at the time of presentation was significant for decreased light touch sensation and hyperalgesia to pinprick in a stocking-glove distribution. Motor strength, proprioception, and tendon reflexes were well preserved.

**Figure 1:**
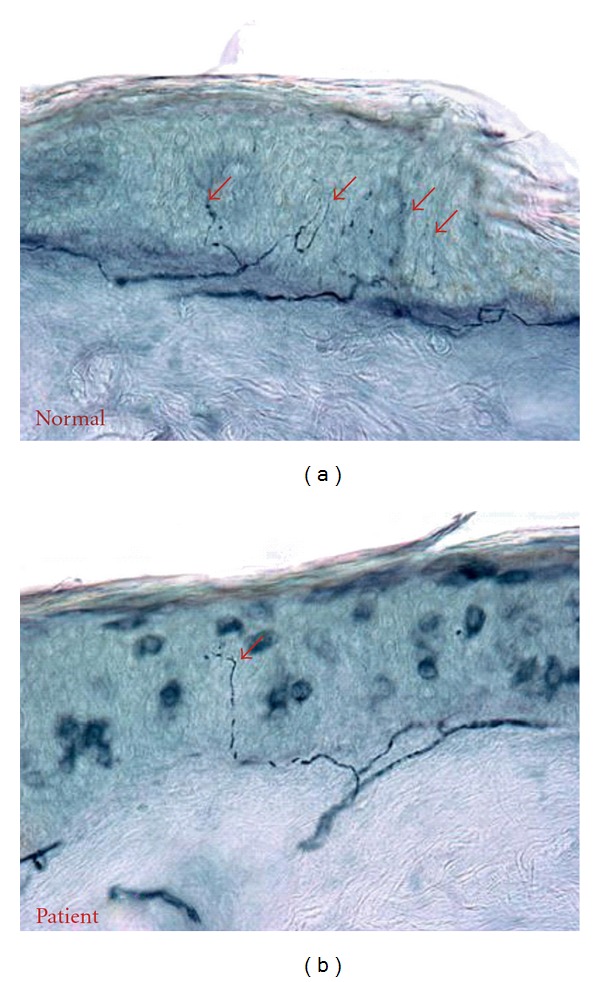
PGP9.5 immunostaining of skin biopsies at the distal leg. The patient with painful diabetic neuropathy (b) showed reduced intraepidermal nerve fibers (red arrow), in comparison to a normal subject (a) who showed many intraepidermal nerve fibers (red arrows).

A nerve conduction study and electromyography was normal showing no evidence of a large fiber peripheral neuropathy or radiculopathy. We further conducted skin biopsies at the distal leg (DL), distal thigh (DT), and proximal thigh (PT) in the Cleveland Clinic Cutaneous Nerve Laboratory and carried out intraepidermal nerve fiber density (IENFD) analysis as previously described [14, 15]. Intraepidermal nerve fiber density was significantly reduced at the distal leg as compared to normal control (Figure 1), which indicates a distal small fiber sensory neuropathy. The neuropathy etiology evaluation was significant for diabetes mellitus as revealed by oral glucose tolerance test with a 2-hour glucose level being 220 mg/dL. ESR, ANA, ENA, rheumatoid factor, ACE level, ANCAs, TSH, free T4, serum and urine immunofixation, folic acid, vitamin B12, RPR, Lyme serology, and heavy metal screen were all unremarkable.

**Figure 2:**
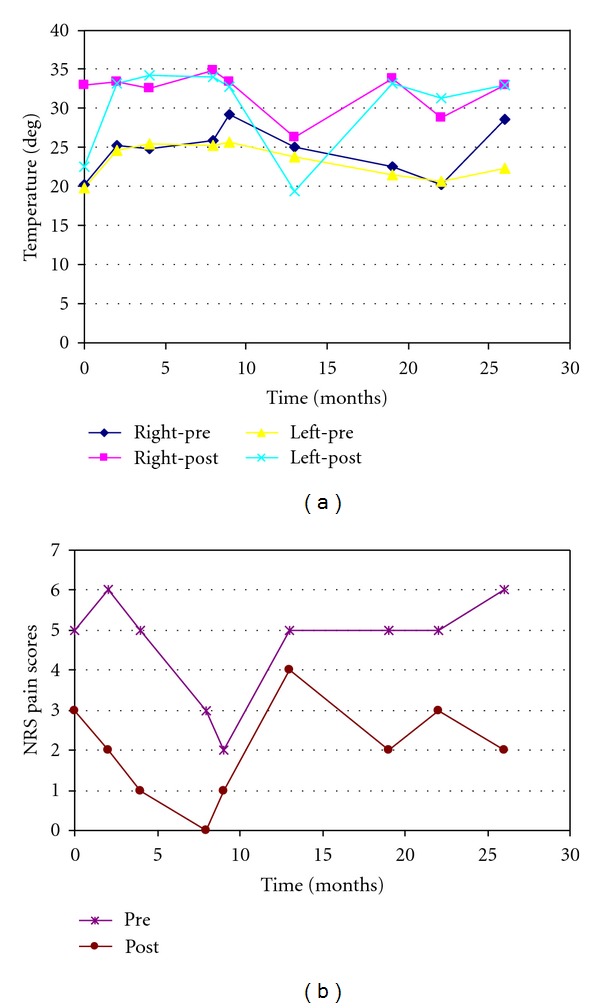
Sympathetic block-induced changes in temperature and NRS pain scores. (a) The peak temperatures, monitored at the plantar surface of the toes with an electronics device, increased significantly in both sides after bilateral lumbar sympathetic blocks (post) compared to the baseline values taken before the blocks (pre). (b) The NRS pain scores, evaluated in the preprocedure room before the blocks (pre) and in the recovery room after the block (post), decreased significantly after each lumbar sympathetic block.

He was diagnosed with painful diabetic small fiber sensory neuropathy and started on Glucophage. In terms of his neuropathic pain control, however, he had failed to respond to multiple pain medications, including Topiramate, Oxcarbazepine, Duloxetine, Amitriptyline, Darvocet, Tramadol, and Lidocaine transdermal patches. Gabapentin only offered minimal pain relief. Given the unsatisfactory outcomes of conservative treatment, we decided to try a lumbar sympathetic block for his pain and allodynia in the low extremities. We used the classic approach for bilateral lumbar sympathetic block at the level of L3 under fluoroscopic guidance and injected a mixture of 12 mL of 1% Lidocaine and 20 mg Triamcinolone on each side. The temperatures, monitored in the plantar surface of the big toes (LS 14000 Temperature Monitor and Skin Temperature Probe, NovaMed, NY, USA), increased significantly, and NRS pain scores decreased substantially after the procedure as shown in Table 1 (initial block) and Figure 2.

At his 2-month followup, the patient reported “excellent” relief of his foot pain bilaterally for over 6 weeks, but became more cognizant of his thoracic and hand pain. A decision was made to proceed with a repeat lumbar sympathetic block to further enhance the pain relief. Subsequent temperature and pain score changes were again noted as shown in Table 1 (2 months) and Figure 2. Management of his thoracic pain was further considered at the following office visit. At a follow-up visit 4 weeks later, the patient again reported “significant, sustained” relief of his bilateral foot pain. He felt “dramatic” improvement of the “coldness and tingling pain” with the first injection with sustained relief that was further enhanced with the second procedure.

At this point, his chief complaint became the “hypersensitive, pins and needles, burning” thoracic and abdominal pain that was described as occupying a discrete antero-/posteroarea of his thorax, roughly covering the T6 to the L1 dermatomes. Physical examination, as previously described, was significant only for bilateral lower extremity stocking-distribution decrease in sensation to light touch and pinprick. He returned 2 weeks later for a T8 bilateral paravertebral sympathetic nerve block, which was performed in the classic fashion, under fluoroscopic guidance. A solution of 1% Lidocaine 10 mL and 20 mg Triamcinolone was injected on each side. The patient was maintained on the same doses of Gabapentin and Tramadol. Upon his return to the clinic 4 weeks later, he reported “70%” relief of his thoracic pain following his T8 paravertebral block. By this point, his bilateral foot pain was 5 on the NRS three months following his second lumbar sympathetic block; therefore, a third bilateral lumbar sympathetic block was performed (Table 1, 4 months). Pain medications remained the same, and the patient was followed up in three months.

Over the next two years, the patient continued to have lower extremity neuropathic pain as described above with good, sustained pain relief after each of subsequent lumbar sympathetic blocks. The intervals of each block are shown in Table 1 (Figure 2) with the corresponding temperature changes and the NRS pre- and postblock pain scores. He additionally underwent two thoracic paravertebral blocks at the T6 level in April and May of 2010 with good relief following each procedure. The remainder of his clinical course was uneventful.

## 3. Discussion

We observed that lumbar and thoracic sympathetic nerve blocks significantly improved the circulations and reduced neuropathic pain in this patient with diabetic small fiber sensory neuropathy. The analgesic effects are reproducible upon repeated blocks and are long-lasting (sustained 2–4 months after each block). These observations support the notion that the sympathetic nervous system plays a critical role in the pathophysiological mechanisms of painful diabetic polyneuropathy. This case report thus provides the first clinical evidence supporting the notion that the pain in diabetic neuropathy may be sympathetically mediated to a significant extent that has not been recognized previously. Given the sustained pain relief after each sympathetic block, the sympathetic nervous system may prove to be an important therapeutic target of pharmacological and interventional treatments for painful diabetic neuropathy.

Painful diabetic neuropathy is a relatively common medical condition, which can predominantly affect small sensory nerve fibers [16]. Glucose dysmetabolism, including diabetes and prediabetes, is present in about 1/3 of patients with painful sensory neuropathy and nearly 50% of otherwise idiopathic small fiber neuropathy [17–20]. Neuropathic pain can be the presenting symptom of diabetes, as seen in this patient. It is one of the most distressing symptoms of diabetic polyneuropathy and the main reason for seeking medical attention. Typical of painful diabetic neuropathy sufferers, this patient experienced a progressive buildup of unpleasant sensory symptoms that include tingling or “pins and needles” (paresthesia), and/or pain characteristic of burning, shooting (like “electric shock” down the legs), lancinating (stabbing or knifelike), and deep aching quality. The patient also developed allodynia and hyperalgesia. Furthermore, the patient experienced pain extended from the lower limbs to the upper limbs and trunk and face, as sometimes seen in advanced cases. Consequently, the patient suffered disturbed sleep and depressive mood after multiple failures to pharmacological treatments.

The patient's neuropathy is consistent with small fiber sensory neuropathy based on his clinical features and skin biopsy findings. Additionally, the patient presented with thoracic and abdominal pain in a dermatomal distribution from T6 to L1 bilaterally. This form of pain can be seen in diabetic peripheral neuropathy although it is not very common [21, 22]. Because the pain in his feet and trunk was most debilitating, we targeted these areas by performing lumbar and thoracic sympathetic blocks. While lumbar sympathetic blocks have been widely used in treating patients with chronic pain conditions in the lower limbs such as complex regional pain syndromes [23–25], paravertebral blocks have predominantly been used for surgical and acute pain management [26]. We utilized the combination of the two blocks and successfully maintained control of the pain in both his legs and trunk for more than 2 years. The novel application of both techniques may thus represent a valuable addition to the armamentarium of interventional pain management.

Although the mechanism of how a mixture of a local anesthetic and a steroid medication can produce long-lasting pain relief is not completely understood as in the case of epidural steroid injections for radicular pain [27], it has been shown that steroids can block nociceptive input. Corticosteroids suppress discharges in chronic neuromas and prevent ectopic discharge in experimental neuromas, likely through a direct action on cell membrane [28]. The application of methylprednisolone has been shown to block C-fibers but not A*β* fibers [29]. Damaged nerve fibers often have high accumulation of expressed sodium channels that are particularly sensitive to local anesthetics such as Lidocaine [30]. Therefore, the sympathetic blocks with local anesthetics and steroids may provide pain relief through similar pharmacological actions on nociceptive fibers. Alternatively, the blocks may reduce sympathetic outflow and circulating norepinephrine, thereby diminishing alpha receptor stimulation of injured peripheral nerve fibers [11]. A third explanation for the pain relief may be due to a sympathectomy-induced improvement in microvascular circulation as indicated by the dramatic increase in temperature of the toes after the blocks (Figure 2), relieving ischemia of nociceptors. It has been recognized that, in addition to a direct toxic effect of glucose on nerve cells, the damage of the nerve structures is accompanied by a microvascular dysfunction, which damages the vasa nervorum. The latter is secondary to the oxidative stress caused by hyperglycemia and other metabolic/homeostatic disorders. This is consistent with the fact that neuropathy and neuropathic pain occur more often in patients whose diabetes is chronically poorly controlled and who also have other cardiovascular risk factors such as hypertension and hyperlipidemia.

While recognizing the limitations of a case study, the findings are evidently intriguing and justify further large-scale studies. Given the inefficiency of current treatment options [7, 31], search for new therapeutic modalities is particularly needed. Interventions targeting at the sympathetic nervous system and its receptors are likely to represent a novel direction of effective therapy.

## Figures and Tables

**Table 1:** Sympathetic block-induced changes in temperature and NRS pain score.

Number of blocks (months after initial block)	Temperature				NRS	
	Right toe		Left toe		Pre	Post
	Pre	Post	Pre	Post		
1 Initial	20.2	33.0	19.7	22.6	5	3
2 (2)	25.3	33.4	24.5	33.2	6	2
3 (4)	24.8	32.6	25.4	34.2	5	1
4 (8)	25.9	34.8	25.2	33.9	3	0
5 (9)	29.2	33.4	25.7	32.7	2	1
6 (13)	24.9	26.3	23.7	19.4	5	4
7 (19)	22.5	33.7	21.5	33.2	5	2
8 (22)	20.2	28.7	20.6	31.3	5	3
9 (26)	28.5	33.0	22.2	33.0	6	2
Average	24.6	32.1	23.2	30.4	4.7	2
